# The Impact of Renal Dysfunction on the Long Term Clinical Outcomes of Diabetic Patients Undergoing Percutaneous Coronary Intervention in the Drug-Eluting Stent Era

**DOI:** 10.1371/journal.pone.0141846

**Published:** 2016-01-05

**Authors:** Ki Hong Choi, Jeong Hoon Yang, Ji Hwan Kim, Young Bin Song, Joo-Yong Hahn, Jin-Ho Choi, Hyeon-Cheol Gwon, Sang Hoon Lee, Seung-Hyuk Choi

**Affiliations:** 1 Division of Cardiology, Department of Medicine, Cardiac and Vascular Center, Samsung Medical Center, Sungkyunkwan University School of Medicine, Seoul, Republic of Korea; 2 Department of Critical Care Medicine, Samsung Medical Center, Sungkyunkwan University School of Medicine, Seoul, Republic of Korea; 3 Department of Emergency Medicine, Samsung Medical Center, Sungkyunkwan University School of Medicine, Seoul, Republic of Korea; University of Hull, UNITED KINGDOM

## Abstract

**Background:**

Limited data are available regarding the association between renal dysfunction and clinical outcomes in diabetic patients undergoing percutaneous coronary intervention (PCI) in the drug-eluting stent (DES) era.

**Methods:**

Between March 2003 and December 2010, 2,181 diabetic patients were enrolled in a single-center registry. We divided diabetic patients into a renal dysfunction group (n = 518) and a non-renal dysfunction group (n = 1,663) according to a baseline estimated glomerular filtration rate <60 mL/min/1.73 m^2^. Propensity score matching analysis was also performed. The primary outcome was cardiac death.

**Results:**

The median follow-up duration was 48 months. The rate of cardiac death was higher in the renal dysfunction group than in the non-renal dysfunction group (14.3% vs. 3.0%, adjusted hazard ratio [HR] 3.63, 95% confidence interval [CI] 2.47 to 5.35, p<0.001). Similarly, the incidence of stent thrombosis was significantly higher in the renal dysfunction group than in the non-renal dysfunction group (4.1% vs. 1.4%, adjusted HR 1.90, 95% CI 1.02 to 3.56, p = 0.04). After 1:1 propensity score matching (502 pairs), patients with renal dysfunction still had a higher rate of cardiac death (13.3% vs. 4.8%, HR 2.58, 95% CI 1.52 to 4.38, p<0.001) although there was no significant difference in the rate of stent thrombosis (4.0% vs. 2.8%, HR 1.31, 95% CI 0.64 to 2.69, p = 0.47).

**Conclusions:**

Renal dysfunction is associated with long-term mortality for diabetic patients undergoing PCI in the DES era.

## Introduction

Diabetes mellitus is regarded as a coronary heart disease risk equivalent as defined by the US National Cholesterol Education Program Adult Treatment Panel III guidelines and is the leading cause of chronic kidney disease (CKD) [1–3]. Several studies have demonstrated that patients with renal dysfunction have an increased risk of adverse clinical outcomes after percutaneous coronary intervention (PCI), including stent thrombosis [4–9]. Renal dysfunction was shown to be an independent predictor of adverse cardiovascular events among diabetic patients undergoing PCI before the introduction of drug-eluting stents (DES) [10–13]. After the development of DES, PCI was expected to improve survival rates in patients with renal dysfunction and prevent restenosis, which is accelerated by atherosclerosis [14]. However, the association between renal dysfunction and long-term cardiovascular outcomes of diabetic patients has not been fully elucidated in the DES era. Therefore, the aim of this study was to determine the association between renal dysfunction and long-term clinical outcomes for diabetic patients who undergo PCI with DES.

## Methods

### Study population

This was a retrospective, single-center, observational study. Between March 2003 and December 2010, a total of 2,187 consecutive diabetic patients who underwent PCI with DES were recruited from the cardiovascular catheterization databases of Samsung Medical Center, Seoul, Korea. Six patients who did not have data on creatinine levels were excluded. Baseline characteristics, angiographic and procedural data, and outcome data were prospectively recorded by PCI registry research coordinators. Additional information was obtained from medical records and telephone interviews if necessary. The requirement for informed consent of the individual patients was waived given the retrospective nature of the study by the Institutional Review Board of the Samsung Medical Center.

### Percutaneous coronary intervention procedure

Coronary interventions were performed according to current standard procedural guidelines. All patients received loading doses of aspirin (300 mg) and clopidogrel (300–600 mg) before coronary intervention unless they had previously received these antiplatelet medications. Anticoagulation therapy during PCI was performed according to the current practice guidelines stipulated by the Korean Society of Interventional Cardiology. The treatment strategy, duration of clopidogrel use, and the use of glycoprotein IIb/IIIa receptor inhibitors or intravascular ultrasound were all performed according to the operator’s discretion. Drug-eluting stents were used without restriction.

### Definitions and Outcomes

Diabetes mellitus was defined as a fasting glucose level >126 mg/dl or glycated hemoglobin A_1c_ concentration greater than 6.5% as assessed at least once, or the current use of oral hypoglycemic agents or insulin. Renal dysfunction was assessed based on a baseline estimated glomerular filtration rate <60 mL/min/1.73m^2^ according to the Modification of Diet in Renal Disease (MDRD) equation [15, 16]. All-cause death was defined as any post-procedure death during follow up and was considered cardiac unless a definite non-cardiac cause was established. Recurrent myocardial infarction (MI) was defined as the recurrence of ischemic symptoms or electrocardiographic changes accompanied by elevated cardiac enzymes. Any revascularization was defined as revascularization of either target or non-target vessels with PCI or bypass graft surgery. Stent thrombosis (ST) was defined using the definitions of the Academic Research Consortium as definite, probable, or possible stent thrombosis [17]. The timing of ST was stratified as early (within 1 month after index procedure), late (between 1 month and 1 year), or very late (after 1 year).

Diabetic patients were divided into two groups according to their baseline estimated glomerular filtration rate (GFR) (<60 or ≥60 ml/min/1.73m^2^). The primary outcome was cardiac death during follow-up. Secondary outcomes included all-cause death, myocardial infarction, ST, and major adverse cardiac and cerebrovascular events (MACCE) consisting of cardiac death, MI, ST, any revascularization, and cerebrovascular events (CVA). Among diabetic patients with renal dysfunction, additional analysis was performed to determine whether duration of dual antiplatelet therapy affects the incidence of ST.

### Statistical analysis

Continuous variables were compared using the *t*-test or Wilcoxon rank-sum test when applicable. Categorical data were tested using the chi-squared test or Fisher’s exact test, as appropriate. Event-free survival was evaluated by Kaplan-Meier analyses and significance level was assessed with the log-rank test. In multivariable models, covariates that were either statistically significant on univariate analysis or clinically relevant were considered candidate variables. To assess the association of renal dysfunction with clinical outcomes, we used a Cox proportional hazards model adjusted for age, sex, hypertension, smoking status, history of PCI, previous MI, history of CVA, multi-vessel disease, left anterior descending artery involvement, and presence of acute coronary syndrome. Propensity score matching was performed in order to reduce selection bias and potential confounding factors. A full nonparsimonious model was developed that included all variables listed in Table 1 with the exception of creatinine level. After propensity score matching, model fit and predictive power were assessed using the Hosmer-Lemeshow test and c-statistic. Cox regression analysis using pairs matched by a greedy algorithm and the nearest available pair-matching method among patients with an individual propensity score was also performed to evaluate the reduction in outcomes risk. The covariate balance achieved by matching was assessed by calculating the absolute standardized mean differences between two groups. Continuous variables were compared with the paired t test or Wilcoxon signed-ranked test, as appropriate. Categorical variables were compared with the McNemar’s or Bowker’s test of symmetry, as appropriate. Cumulative incidence rates of individual clinical outcomes and composite outcomes were estimated by the Kaplan-Meier method and compared by the paired Prentice-Wilcoxon test. Statistical analyses were performed with SAS 9.2 (SAS Institute Inc., Cary, NC, USA). All tests were two-tailed and p <0.05 was considered statistically significant.

**Table 1:** Baseline clinical and angiographic characteristics stratified according to renal function.

	Total Population				Propensity-Matched Population			
	Non-RD (n = 1663)	RD (n = 518)	p value	Standardized difference	Non-RD (n = 502)	RD (n = 502)	p value	Standardized difference
Age (yr)	62.68±9.9	69.21±9.5	<0.001	68.3	68.48±8.5	68.84±9.4	0.53	3.8
Male (%)	1232(74.1)	327(63.1)	<0.001	22.7	325(64.7)	319(63.5)	0.703	2.5
Current smoker (%)	340(20.5)	62(12.0)	<0.001	-26.1	62(12.4)	62(12.4)	>0.99	0
Body mass index (kg/m^2^)	24.75±2.9	24.31±3.4	0.01	-12.4	24.22±2.9	24.38±3.4	0.40	4.9
Hypertension (%)	1077(64.8)	438(84.6)	<0.001	54.7	416(82.9)	422(84.1)	0.61	3.3
Dyslipidemia (%)	549(33.0)	155(29.9)	0.20	-6.7	147(29.3)	153(30.5)	0.68	2.6
Insulin treatment (%)	38(2.3)	31(6.0)	<0.001	15.6	30(6.0)	25(5.0)	0.49	-4.2
Prior PCI (%)	261(15.7)	103(19.9)	0.03	10.5	88(17.5)	99(19.7)	0.37	5.5
Prior bypass surgery (%)	61(3.7)	29(5.6)	0.06	8.4	28(5.6)	28(5.6)	>0.99	0
Prior MI (%)	354(21.3)	144(27.8)	0.003	14.5	136(27.1)	135(26.9)	0.94	-0.4
Prior CVA (%)	103(6.2)	54(10.4)	0.002	13.8	53(10.6)	53(10.6)	>0.99	0
Clinical presentation			0.45				0.53	
Stable angina (%)	961(57.8)	296(57.1)		-1.3	286(57.0)	291(58.0)		2.0
Unstable angina (%)	308(18.5)	87(16.8)		-4.6	94(18.7)	81(16.1)		-6.9
MI (%)	394(23.7)	135(26.1)		5.4	122(24.3)	130(25.9)		3.6
Hemoglobin A1c (%)	7.66±1.6	7.76±1.8	0.33	4.7	7.78±1.6	7.73±1.8	0.69	-2.5
Creatinine (mg/dL)	0.88±0.1	2.32±2.1	<0.001		0.88±1.7	2.33±2.1	<0.001	
MVD	1099(66.1)	391(75.5)	<0.001	21.8	380(75.7)	375(74.7)	0.72	-2.3
Involved vessel			0.06				0.75	
RCA	552(33.2)	169(32.6)			164(32.7)	165(32.9)		
LAD	737(44.3)	229(44.2)		-0.2	230(45.8)	224(44.6)		-2.4
LCX	305(18.3)	84(16.2)		-5.7	79(15.7)	79(15.7)		0
Left main	58(3.5)	33(6.4)		11.8	26(5.2)	31(6.2)		4.1
Graft	11(0.7)	3(0.6)		-1.1	3(0.6)	3(0.6)		0
No. of treated vessel	1.55±0.8	1.62±0.9	0.16	7.0	1.63±0.9	1.62±0.9	0.78	-1.8

Data are presented as mean ± standard deviation or n (%).

CVA = cerebrovascular accident(s); LAD = left anterior descending artery; LCX = left circumflex artery; MI = myocardial infarction; MVD = multi-vessel disease; PCI = percutaneous coronary intervention; RCA = right coronary artery; RD = renal dysfunction

## Results

### Baseline and angiographic characteristics

#### Overall population

A total of 2,181 consecutive diabetic patients who underwent PCI with DES were analyzed in this study. Among the patients, 518 (23.8%) had renal dysfunction and 1,663 (76.2%) had preserved renal function. Baseline clinical and angiographic characteristics according to estimated GFR are described in Table 1. Patients with renal dysfunction were more likely to be women, older, and have a lower body mass index, lower prevalence of smoking, and a higher prevalence of hypertension, insulin treatment rates, prior PCI or bypass surgery, previous MI, CVA, peripheral vascular disease, and higher serum creatinine level. There were no significant differences in clinical presentation and glycated hemoglobin A_1c_. Multi-vessel disease was more common in the renal dysfunction group, but the vessel involved and the number of treated vessels was similar between the two groups.

#### Propensity-matched population

After performing propensity score matching for the entire population, a total of 502 matched pairs of patients were obtained (Table 1). The c-statistic for the propensity score model was 0.73, which indicates good discrimination (Hosmer-Lemeshow goodness of fit p = 0.98). There were no significant differences in baseline clinical and angiographic characteristics for the propensity-matched subjects, with the exception of creatinine level. All variables had acceptable standardized mean differences.

### Clinical Outcomes

#### Overall population

Follow-up data were available for 2,174 patients (99.6%) with a median follow-up of 48 months (interquartile range 27 to 70). There were a total of 124 (5.7%) cardiac deaths during the entire follow up period. The incidence of cardiac death was significantly higher in the renal dysfunction group than the non-renal dysfunction group (renal dysfunction group versus non-renal dysfunction group, 14.3% vs. 3.0%, adjusted hazard ratio [HR] 3.63, 95% confidence interval [CI] 2.47 to 5.35, p<0.001) (Table 2). Similarly, patients with renal dysfunction had significantly higher rates of all-cause death, MI, CVA, and MACCE than those without renal dysfunction (Table 2). However, the incidence of revascularization did not differ between the two groups. The rate of ST was significantly higher in the renal dysfunction group (4.1% vs. 1.4%; adjusted HR 1.90, 95% CI 1.02 to 3.55, p = 0.044) (Table 2). Definitive or probable ST developed in 45 patients (2.1%) during follow up: early ST in 20 patients (0.9%), late ST in 12 patients (0.6%), and very late ST in 13 patients (0.6%). ST was the cause of death in 25 patients during the study period.

**Table 2:** Clinical outcomes in RD group compared with non-RD group in all patients during follow-up period.

Total population (n = 2181)	Non-RD group (n = 1663)	RD group (n = 518)	Unadjusted HR (95% CI)	p value	Adjusted HR (95% CI) ^a^	p value
Cardiac death	50 (3.0)	74 (14.3)	5.32 (3.71–7.62)	<0.001	3.63 (2.47–5.35)	<0.001
All-cause mortality	114 (6.9)	145 (28.0)	4.64 (3.63–5.94)	<0.001	3.05 (2.34–3.97)	<0.001
Myocardial infarction	55 (3.3)	42 (8.1)	2.80 (1.87–4.19)	<0.001	2.03 (1.32–3.12)	0.001
Any revascularization	231 (13.9)	73 (14.1)	1.15 (0.89–1.50)	0.29	1.05 (0.80–1.38)	0.73
Cerebrovascular accident	72 (4.3)	33 (6.4)	1.66 (1.10–2.51)	0.02	1.17 (0.76–1.81)	0.49
Stent thrombosis^b^	24 (1.4)	21 (4.1)	3.00 (1.67–5.40)	<0.001	1.90 (1.02–3.56)	0.04
MACCE	332 (20.0)	167 (32.2)	1.85 (1.53–2.23)	<0.001	1.53 (1.26–1.87)	<0.001

CI = confidence interval; HR = hazard ratio; MACCE = major cardiac and cerebrovascular event(s); other abbreviations as in Table 1.

^a^Adjusted covariates included age, sex, hypertension, current smoker, history of PCI, history of MI, history of CVA, multi-vessel disease, LAD involvement, and presence of acute coronary syndrome.

^b^Stent thrombosis was defined as definite or probable.

#### Propensity-matched population

**Fig 1:**
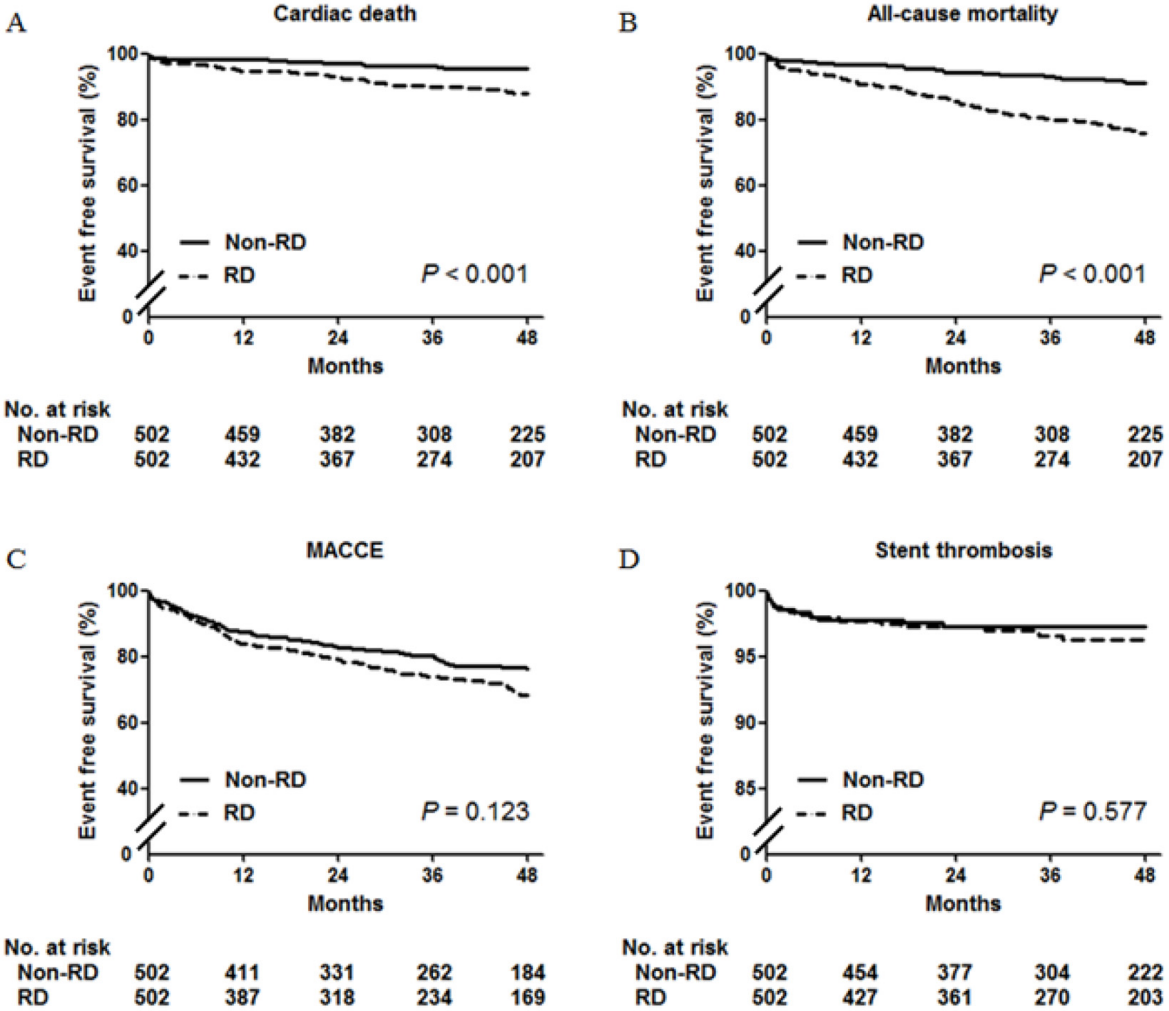
Kaplan-Meier curves for propensity-matched patients according to presence of renal dysfunction. Kaplan-Meier curves in propensity-matched patients for cardiac death (A), all-cause death (B), MACCE (C), and stent thrombosis (D) in the non- renal dysfunction (solid line) group versus renal dysfunction (dashed line) group. MACCE = major adverse cardiac and cerebrovascular event(s); RD = renal dysfunction.

The median follow-up duration was 42 months (interquartile range 23 to 65). There were 91 (9.1%) events of cardiac death during the entire follow up period. After 1:1 propensity-score matching, the renal dysfunction group still had a higher rate of cardiac death (13.3% vs. 4.8%, HR 2.58, 95% CI 1.52 to 4.38, p<0.001) (Table 3, Fig 1A) and all-cause death (26.9% vs. 10.8%, HR 2.91, 95% CI 1.97 to 4.30, p<0.001) (Table 3, Fig 1B). However, there were no significant differences between the two groups in the incidence of definite or probable ST (4.0% vs. 2.8%, HR 1.31, 95% CI 0.64 to 2.69, p = 0.467) and MACCE (31.9% vs. 25.1%, HR 1.28, 95% CI 0.97 to 1.70, p = 0.086) (Table 3, Fig 1C and 1D).

**Table 3:** Clinical outcomes during follow up period in RD group compared with non-RD group in a propensity matched population.

Propensity matched population (n = 1,004)	Non-RD group (n = 502)	RD group (n = 502)	Hazard ratio (95% CI)	p value
Cardiac death	24 (4.8)	67 (13.3)	2.58 (1.52–4.38)	<0.001
All-cause mortality	54 (10.8)	135 (26.9)	2.91 (1.97–4.30)	<0.001
Myocardial infarction	29 (5.8)	39 (7.8)	1.39 (0.81–2.38)	0.23
Any revascularization	78 (15.5)	72 (14.3)	1.04 (0.71–1.53)	0.84
Cerebrovascular accident	30 (6.0)	33 (6.6)	0.96 (0.53–1.74)	0.88
Stent thrombosis	14 (2.8)	20 (4.0)	1.31 (0.64–2.69)	0.47
MACCE	126 (25.1)	160 (31.9)	1.28 (0.97–1.70)	0.09

Abbreviations as in Tables 1 and 2.

### Duration of dual antiplatelet and stent thrombosis

Diabetic patients with renal dysfunction were analyzed based on whether they had undergone less or greater than 12 months of dual antiplatelet therapy. Patients who died within a year of undergoing PCI were excluded from the analysis. Among those included, there were 124 patients who had been on less than 12 months of dual antiplatelet therapy of whom 4 had definitive or probable ST (3.2%), whereas there were 339 patients who had been on greater than 12 months of therapy of whom 8 had ST (2.4%). The difference between the two groups was not statistically significant (HR 1.20, 95% CI 0.36 to 4.00, p = 0.768).

### Clinical outcomes according to the grade of renal insufficiency

We performed additional analyses to evaluate the association of a decrease in GFR with the rates of cardiac death and stent thrombosis. Study patients were divided into four groups according to their baseline estimated GFR (>90, 89–60, 59–30, and <30 ml/min/1.73m2). Table 4 shows cumulative clinical outcomes according to the level of GFR. There were no significant differences in the rates of cardiac death and ST between normal renal function (GFR > 90) and mild renal insufficiency (GFR 89 to 60) groups. However, moderate or severe renal insufficiency groups (GFR 59 to 30 and <30) had higher rates of cardiac death compared with normal renal function group. Severe renal insufficiency group (GFR <30) had a trend toward a high incidence in ST (6.9%) compared with normal renal function group (2.0%).

**Table 4:** Clinical outcomes according to the grade of renal insufficiency.

Subdivision of GFR (n)	Cardiac death				Stent thrombosis^b^			
	n (%)	Adjusted HR^a^	95% CI	P value	n (%)	Adjusted HR	95% CI	P value
GFR > 90 (n = 494)	19 (3.8)	1			10 (2.0)	1		
GFR 89 to 60 (n = 1169)	31 (2.7)	0.56	0.32 to 1.00	0.05	14 (1.2)	0.59	0.26 to 1.34	0.20
GFR 59 to 30 (n = 359)	43 (12.0)	1.96	1.10 to 3.51	0.02	10 (2.8)	0.92	0.36 to 2.34	0.85
GFR < 30 (n = 159)	31 (19.5)	3.91	2.13 to 7.19	<0.001	11 (6.9)	2.39	0.97 to 5.91	0.06

GFR = glomerular filtration rate; other abbreviations as in Table 2.

^a^Adjusted covariates included age, sex, hypertension, current smoker, history of PCI, history of MI, history of CVA, multi-vessel disease, LAD involvement, and presence of acute coronary syndrome.

^b^Stent thrombosis was defined as definite or probable.

## Discussion

In the present study, we investigated the association between renal dysfunction and long-term cardiovascular outcomes in diabetic patients undergoing PCI with DES using a single-center registry. The major findings of this study were that renal dysfunction was associated with higher rates of cardiovascular events in diabetes patients after PCI with DES. In particular, the association of renal dysfunction with a higher incidence of cardiac death was maintained in propensity-matched populations. In addition, definite or probable stent thrombosis was numerically higher in the renal dysfunction group than the non- renal dysfunction group, there was no statistically difference between the two groups.

For the pre-DES era, a previous study showed that diabetic patients with CKD had an almost 2-fold higher rate of all-cause death and 1.5-fold higher rate of MACCE (death, MI, bypass surgery, or target lesion PCI) compared with those without CKD [10]. Similarly, in our study of patients undergoing PCI with DES, patients with renal dysfunction had a 2- to 3-fold higher rate of cardiac death, all-cause death, and MI compared with those without renal dysfunction after performing propensity-score matching; and the rate of MACCE was almost 1.5-fold higher in the renal dysfunction group compared with the non-renal dysfunction group. These findings suggest that the association of renal dysfunction with worse clinical outcomes in diabetic patients undergoing PCI was maintained in the DES era even though the introduction of DES has dramatically reduced the restenosis rate.

CKD is not defined as a coronary risk equivalent in the current guideline due to insufficient data [18]. However, a recent large cohort study showed that the diabetes and CKD group had a greater relative risk of MI and all-cause death than the previous MI group [19]. In addition, CKD is associated with an increased bleeding risk and a greater risk of thrombotic complications, including stent thrombosis [7–9, 20–22]. Moreover, the presence of a low platelet response to clopidogrel is related to worse cardiovascular outcomes after PCI in patients with CKD [23]. However, comparison of ST between renal dysfunction and non-renal dysfunction groups among diabetic patients has been not fully evaluated in the DES era. In this study, we demonstrated that the risk of ST (definitive or probable) in the DES era is increased almost 2-fold in diabetic patients with renal dysfunction (4.1%) compared with those without renal dysfunction (1.4%). This finding is in agreement with a previous study indicating that impaired renal function is related to the reduced anti-platelet effects of clopidogrel in diabetes patients undergoing PCI [24]. After performing propensity-score matching, although the absolute incidence of stent thrombosis was higher in the renal dysfunction group than in the non-renal dysfunction group, no statistically significant difference was found between the two groups. The discrepancy between multivariate analysis and propensity score matching may be due to the very low incidence of ST. Further prospective multi-center studies with a large study population would be helpful to confirm the results of our study.

Dual antiplatelet therapy improves clinical outcomes after PCI through reductions in both ST and MI not related to ST [25]. Current practice guidelines recommend at least 12 months of dual antiplatelet therapy in patients with acute coronary syndrome undergoing PCI with DES [26]. However, optimal duration of dual antiplatelet use has been subject to debate. Though it seems logical to presume benefit to longer duration of dual antiplatelet therapy in diabetic patients with renal dysfunction due to their increased risk of thrombosis and resistance to P2Y12 inhibitors, among diabetic patients with renal dysfunction in the present study, dual antiplatelet therapy duration after PCI (less than 12 months vs. more than 12 months) did not show any statistically significant difference on the incidence of ST. It is uncertain why there were no significant differences between the two groups in the incidences of ST. Accordingly, a large randomized controlled trial is needed to further elucidate the optimal duration of dual antiplatelet therapy in diabetic patients with renal dysfunction.

### Study limitations

This study had several limitations. First, it was an observational study, which may have affected the results because of confounding factors. In addition, the selection of treatment strategies was based on the doctor’s preference. Although we performed various risk adjustments for potential confounding factors, including propensity score matching, we were not able to correct for unmeasured variables. Second, in particular, due to the limitations of our data base, we did not have information on why physicians discontinued dual antiplatelet therapy. Third, estimated GFR was assessed by laboratory data from a single time-point and the influence of contrast toxicity on clinical outcomes or laboratory data was not investigated. However, the earliest measured serum creatinine before PCI, which should most accurately represented the patients’ baseline kidney function, was recorded. Finally, our results must be interpreted with caution considering the relatively small sample size and the lack of power to detect differences in ST.

## Conclusion

Renal dysfunction was associated with worse long-term clinical outcomes in diabetic patients undergoing PCI despite the introduction of DES.

## Supporting Information

S1 Dataset Minimal relevant dataset of this study. (CSV)

## Abbreviations

CKD: chronic kidney disease
CVA: cerebrovascular accident(s)
DES: drug eluting stent(s)
GFR: glomerular filtration rate
MI: myocardial infarction
MACCE: major adverse cardiac and cerebrovascular event(s)
PCI: percutaneous coronary intervention
ST: stent thrombosis
